# Food design, nutrition, and innovation

**DOI:** 10.3389/fpubh.2022.1039795

**Published:** 2022-10-24

**Authors:** Fabio Parasecoli

**Affiliations:** Nutrition and Food Studies Department, New York University, New York, NY, United States

**Keywords:** food design, food design thinking, food system, human-centered design, participative design, food communication

In their work, nutritionists and public health professionals are often involved in projects meant to improve the quality of life of individuals and communities, dedicating great efforts to implement them and convince the target populations to embrace them (1). At times, however, the recipients of these initiatives experience them as top-down impositions or bureaucratic nuisances that are disconnected from their everyday lives, needs, priorities, and preferences. These dynamics, which have prompted calls for the application of approaches inspired by nudging (2), may be rooted in difficulties at various phases of the initiative at hand: the way studies and research are structured to identify a problem and propose solutions; the way the implementation of projects emerging from those studies is designed and organized; and the way the initiative is communicated to the public. To avoid these obstacles, literature calls for greater participation and collaboration among the stakeholders involved (3, 4). Furthermore, design and design thinking have been suggested as viable instruments to facilitate innovation (5, 6).

In this short reflection I explore design, and food design in particular, as a possible approach to these matters and as a repository of tools that could be used to improve the research underlying food- and nutrition- related public health initiatives, their implementation, and their popularization. Although my scholarly work is in food studies, I have been collaborating for years with designers to develop projects, conduct research, and brainstorm ideas. This essay is based on these applied experiences, theoretical reflections, and methodological approaches.

It seems appropriate to provide some information about food design, a budding but quickly expanding field of scholarly research and professional practice that is still relatively unknown both within and outside academia. On the occasion of the launch of the now inactive Food Design North America working group, designer Pedro Reissig suggested that the goal of food design is to “improve our relationship with food, individually or collectively, in the most diverse ways and instances. Its actions can relate to the design of food products, materials, practices, environments, systems, processes and experiences” [(7), p. 157–158]. In reality, there is no single definition of what the field is, what includes, and what conversely does not fall under its umbrella, as a recent collection of opinions about food design from professionals and researchers from all around the world indicates (8). This variety of theoretical, methodological, and professional perspectives is reflected in how food design engages with cultural and social issues, the use of objects and spaces, communal wellbeing, and musings about the impact of technology.

As food design is inherently concerned with the future, it can contribute to the transformation of food systems toward greater sustainability, equity, and attention to human needs, including health and nutrition. To design is always to redesign; when is centered on humans, design can draw attention to the material conditions surrounding the stakeholders in any endeavor, besides their practices and discourse. It focuses on the affordances of the objects, spaces, and relations in the environment where projects are supposed to be developed. For this reason, design emphasizes what people actually do rather than what they think or say they do. To implement these tactics, many scholars and professionals that consider themselves as operating within the framework of food design employ a design thinking approach (9). In this iterative, practice-based method, practitioners examine the issue at hand in order to gain insights and generate ideas and strategies, which are then prototyped and tested. This process is iterative: the feedback from tests is leveraged to attain new insights, and so on. The goal is to refine various possible solutions and to select the best one for implementation (10). With health and nutrition as goals, such approaches could positively influence what is in a plate, the plate itself, the environments where the food is consumed, and the multilayered connections that make it part of local, national, and global food systems.

Design tools could be unfamiliar to public health researchers; at the beginning, it would be most effective to establish collaborations with designers that focus on food from the points of view of experiences, services, and systems. Although at times complex, such interactions allow all parties involved to examine familiar issues with fresh eyes, to apply different analytical categories, and to look for solutions in unexpected areas of research and intervention. For instance, in collaborations with food manufacturers, public health researchers may tend to emphasize the nutritional content of a new healthy product, as well on its impact on individual and community wellbeing. That may not be sufficient to make the product interesting or inviting, despite its clear health benefits. Designers may then contribute by finding ways to make the new product more appealing, to improve the users' experience around it, and to make it accessible and easy to use. As public health researchers become more familiar with design tools and approaches, they may start to apply them on their own on the projects they work on.

Design approaches can facilitate communication among all the stakeholders involved in innovation processes. As I already mentioned, design tools can be employed to improve the preliminary research for initiatives that range from scholarly investigations to community-based plans. In a research project about the revaluation of regional and traditional foods in Poland, in 2019 my team and I organized a food design workshop in Warsaw with young chefs between 25 and 35 years of age who were managing their own restaurants. Our goal was to get a better sense of the local culinary landscape and how young chefs thought about innovation, tradition, and health, as traditional Polish food is often considered heavy and unhealthy. Instead of using a focus group, interviews, or observations (all very established methods in ethnography), on that occasion we asked the participants to engage in activities that allowed them not only to reflect on their own prospects, but also to imagine what the future of Polish food could be like. We invited them to design menus, both as individuals and in groups, figure out dish costs, and envision communication strategies with their patrons. This experience contributed to a decisive shift in our research focus, nudging us toward a deeper understanding of the role of the actors we call tastemakers, that is the stakeholders introducing and supporting new approaches to make Polish food more exciting, lighter, healthier, and more cosmopolitan without renouncing its roots (11).

Food design can also support strategies to make information and data about complex phenomena more accessible to the public. This was the goal of a project I developed as part of a team at The New School, in collaboration with the Red Cross Red Crescent Climate Centre, for the Community Based Adaptation to Climate Change (CBA 11) conference in Uganda in June 2017 (12). The idea was to create experiences that could help participants better understand climate change. Some of the concepts included a pizza pie that demonstrated the proportion of clean vs. fossil fuel energy with a perfectly edible and appetizing portion while the remaining two thirds of it were burnt; a dessert with a startling garnish of crickets to prompt the exploration of alternative, environmentally-friendly protein sources; and a range of salad dressings with increasing proportions of spicy North African harissa to show how global warming caused increasing discomfort. Students also proposed experiences that could support Ugandan farmers in grasping the consequences of weather pattern changes on agriculture and food waste by using local ingredients, dishes, and practices. For instance, they outlined a game in which the absence of necessary ingredients, reflecting the effects of climate change on food availability, would force participants to get creative in cooking.

Food design can become a pedagogical device to encourage students' critical engagement with different aspects of their food environment and to stimulate their active participation is food- and nutrition-related initiatives. In a course I taught at the Nutrition and Food Studies Department at New York University, I guided students to investigate the role that design played in shaping the food system, from production to consumption, exploring stakeholders' interactions with designed places, objects, sensorial experiences, as well as ideas, services, and systems. Each reading and assignment was meant to increase students' analytical toolbox to further examine the food landscape around them and to engage with it through transformation and innovation. Through their assignment, students honed their ability to think systemically and strategically by connecting food and design, and by developing proposals for food-related projects. Some of the students have continued working in the design and food design spaces after graduation.

A similar inspiration is behind two museums exhibitions I have contributed to. The first one was *Food: Bigger than the Plate*, which ran from May through October 2019 at the Victoria and Albert Museum in London. As the museum website stated, “from urban farming to gastronomic experiments and synthetic meat, this exhibition brought together the politics and pleasure of food to ask how the collective choices we make can lead to a more sustainable, just and delicious food future” (13). Design played a central role in the project as a way to conceptualize the theme, create narratives to communicate it to the general public, and translate these ideas into objects in a specific physical space. However, design was also offered to audiences as a topic of reflection in itself: what does it mean to design a food system? What could a design approach bring to the way we interact with the food landscape that surrounds us? (14). Design was proposed not only as an analytical framework, but also as a tool to empower visitors to think of themselves as stakeholders that could participate and have a say in local and global processes. This is also the inspiration for the US adaptation of the London exhibition, Food in New York: Bigger than the Plate, which opened in September 2022 at the Museum of the City of New York. In this case, however, the topics are much more focused on New York City and the specific challenges its unique food system faces. The positive feedback in media and in public conversations both exhibitions are receiving suggest their efficacy in making urgent food- and nutrition-related issues visible among the general public and in generating productive conversations.

Food design also comes in handy when working with the food industry. Large corporations are increasingly embarking on innovation projects aimed at better nutrition and greater environmental and social sustainability, even if often marketing and public relations constitute the real motivations behind these initiatives. Moreover, changes can only go so far because executives, managers, and designers have to respond to shareholders. However, research about this kind of food design applications is not easy: experiences in brainstorming and actual transformation processes cannot be publicly shared when proprietary products and business models are covered by non-disclosure agreements, which is often the case. Nonetheless, scholars and professionals who participate in such processes can use the acquired know-how and innovative approaches both in public projects and in teaching students.

The applications of design to food can be extended to apps and distribution networks, which have boomed worldwide during the COVID pandemic, in order to improve the availability and convenience of healthy and nutritious food while paying attention to environmental sustainability and the working conditions of deliverers. Food design approaches can be applied to improve restaurant menus in terms of portion sizes, quality of ingredients, and healthiness while minimizing the impact on customers' enjoyment and respecting the cultural significance of certain foods and dishes. Food design could contribute to rethinking supermarkets, stores, and farmers markets. It could better integrate street food in urban design, improving sellers' material conditions and the healthiness of their offerings. As these experiences and approaches are quite new, it is still early to evaluate the long-term impact of the interventions that are being implemented, often in experimental phases. Regardless of the food system aspects to which food design is applied, to ensure its effectiveness what should remain central is the constant focus on the participation of all stakeholders involved in all phases of new initiatives, from research design to implementation and communication.

## Author contributions

The author confirms being the sole contributor of this work and has approved it for publication.

## Conflict of interest

The author declares that the research was conducted in the absence of any commercial or financial relationships that could be construed as a potential conflict of interest.

## Publisher's note

All claims expressed in this article are solely those of the authors and do not necessarily represent those of their affiliated organizations, or those of the publisher, the editors and the reviewers. Any product that may be evaluated in this article, or claim that may be made by its manufacturer, is not guaranteed or endorsed by the publisher.
